# Supplementary material for: Statins, metformin, and RAS inhibitors did not reduce variceal bleeding risk and mortality in a large, real-life cohort of patients with cirrhosis
Source: PLoS One. 2024 Jun 13;19(6):e0302811. doi: 10.1371/journal.pone.0302811 (PMC11175511; doi:10.1371/journal.pone.0302811)
Supplement: Attachment — Submitted filename: 240325_PFISTERER_MS_EBL and CoMeds_Response letter.docx [file pone.0302811.s001.docx]

Vienna, 24-March 2024

Univ. Prof. PD Dr. Thomas Reiberger

T: +43 (0)1 40400 -65890

F: +43 (0)1 40400 -47350

[**thomas.reiberger@meduniwien.ac.at**](mailto:thomas.reiberger@meduniwien.ac.at)

Cirrhosis (ACLD) Clinic	Viral Hepatitis/HIV Clinic	Hepatic Hemodynamic Lab	Pre/Post Livertransplant Clinic	Vienna General Hospital (AKH Wien)

**Head: Christian-Doppler & HEPEX Lab for Portal Hypertension and Liver Fibrosis at the MedUni Vienna**

Head: LBG clinical research group MOTION at MedUni Vienna

Coordinator of the European Reference Network for Rare Liver Diseases (ERN RARE LIVER) at the Vienna General Hospital (AKH Wien)

Adjunct-PI at the Center for Molecular Medicine (CeMM) of the Austrian Academy of Science (OeAW)

Div. Gastroenterology & Hepatology Department of Medicine III

Medical University of Vienna

Head & Chair: Prof. Dr. Michael Trauner

Spitalgasse 23, 1090 Vienna, Austria
[www.meduniwien.ac.at](http://www.meduniwien.ac.at)

T: +43 (0)1 40400 -47410

F: +43 (0)1 40400 -47350

**To the Editorial Board**

**of PLOS ONE**

**Dear Professors,**

**RE: Revisions “PONE-D-23-35530” “Statins, metformin, and RAS inhibitors did not reduce variceal bleeding risk and mortality in a large, real-life cohort of patients with cirrhosis”**

We would like to express our gratitude for the acceptance and thorough review of our study on bleeding and mortality rates in a large, real-world cohort of patients with cirrhosis that focused on the impact of co-medication with statins, metformin, and RAS inhibitors. We have taken this opportunity to revise our study and considered all valuable comments and suggestions received from the Editors and the Reviewers. We have collected additional data and performed detailed analyses that ultimately, further strengthen our conclusions. Specifically, we have:

- assessed follow-up duration, bleeding events and mortality separately for the primary prophylaxis group and the secondary prophylaxis group, while still comparing the intake of comedication within these subroups.
- utilized a Cox regression model to adjust for Child-Pugh stage and age, showing that the intake of at least one comedication did not significantly affect (re-)bleeding risk or survival outcomes.
- performed additional analyses on the effects of individual comedications on bleeding when added on top of NSBB treatment and found that, in both primary and secondary prophylaxis, the synergistic effect of NSBB and all comeds on decompensation, such as (re-)bleeding, has no significant effect.
- Revised our discussion section by considering the important suggestions pointed out by the reviewers, such as the lack of systematic assessment of ongoing alcohol consumption.

Please find our point-by-point response below. Thank you for considering our revised article for publication in PLOS ONE. With kind regards on behalf of all co-authors,

Dr. Nikolaus Pfisterer	Prof. Dr. Thomas Reiberger

**Comments from the Editorial Board:**

Reviewer 1:

**1) Timepoint of Comparison: In my opinion, the authors should have selected a different timepoint for comparison. At the time of inclusion, patients already exhibited clinically significant portal hypertension, indicated by the presence of high-grade varices or a bleeding event in the secondary prophylaxis group. The consequences of portal hypertension likely overrode the effects of comeds at this stage. Moreover, it would be beneficial to know, how long before baseline these medications were taken. The time from the start of intake to baseline should be included in the statistics or, if unavailable, this should be mentioned in the limitations. A more appropriate timepoint for comparison might be 1 or 2 years after beginning therapy with these comeds. Do the authors possess this data? Perhaps, using this timepoint of analysis, it might not even be necessary to compare between primary and secondary prophylaxis groups.**

We absolutely agree with reviewer 1 that a landmark analysis at certain time points after starting the intake of the comedications of interest (i.e., reflecting cumulative exposure, and more likely the effect on “early portal hypertension” before the development of high-risk varices or bleeding) would be an interesting analysis. However, our study design does not allow to investigate timeperiods and comedication intake before the first endoscopic examination at the study centers, because only this referral triggered the inclusion in our study. Thus, our study can only investigate patients with portal hypertension and varices, including those with previous variceal bleeding (i.e., in primary or secondary bleeding prophylaxis), and we only have data on medication intake and continuation after the first registered endoscopic examination/intervention at one of the two study centers. We thus can only follow the suggestion of the reviewer to discuss this relevant potential impact of comedication before study inclusion, and mention the lack of potential to investigate the cumulative effect of the comedications of interest before the first documentation of varices, i.e. the first endoscopic procedure performed at one of our study centers. We will certainly acknowledge this as a limitation in our study.

We included the interesting idea in our discussion section:

*“...However, future studies using well-matched control groups regarding comorbidities – ideally of randomized design, should explore the effects of statins, metformin, and RASi on clinical endpoints in patients with cirrhosis and portal hypertension. Furthermore, an investigation of the impact of statins, metformin, and RASi specifically in patients with liver cirrhosis and “early” portal hypertension, e.g. before varices have bled would be an interesting focus for a separate study…”*

*“…Another important limitation is the missing information on cumulative drug exposure prior to study inclusion …”*

**2) Comparison Between Groups: In the primary prophylaxis group, 13.2% of patients experienced a first bleeding event, and 36.8% died. In the secondary prophylaxis group, 30% had further bleeding events, and 49.1% died. Is the comparison significant between the two groups? Did the authors compare the number of patients with comeds vs. no-comeds in the respective groups? The authors present results only within the two groups.**

Thank you for these suggestions. In *Table-R1* we compared bleeding events and mortality between the primary prophylaxis group and the secondary prophylaxis group. As the reviewer mentioned, in the primary prophylaxis group, 13.2% of patients experienced a first bleeding event, and 36.8% died. In contrast, in the secondary prophylaxis group, 29.2% had further bleeding events, and 47.5% died.

In *Table-R1*, the median follow-up duration (evaluated using the Mann-Whitney U test) was similar between groups, with primary prophylaxis at 26.8 months (interquartile range: IQR 37.9) and secondary prophylaxis at 29.2 months (IQR 56.7, p=0.782).

However, statistically significant differences were observed in bleeding and mortality outcomes, evaluated using Fisher's exact test: The incidence of bleeding during follow-up was significantly higher in the secondary prophylaxis group at 29.2%, compared to 13.2% in the primary prophylaxis group (p<0.001). Similarly, mortality rates during follow-up were elevated in the secondary prophylaxis group at 47.5%, versus 36.8% in the primary group (p=0.001). Regarding the one-year outcomes for bleeding and mortality (p=0.012 and p=0.026, respectively), this underscores the distinct risk profiles and outcomes between patients undergoing primary versus secondary prophylaxis.

Furthermore, on reviewer 1’s recommendations, we also analyzed the distribution of patients receiving at least one comedication (such as a statin, metformin, and/or RAS inhibitor) between the primary and secondary prophylaxis groups (*see Table-R1*). In the primary prophylaxis group, 22.7% of patients were on at least one comedication, whereas in the secondary prophylaxis group, only 14.7% had at least one medication of interest. The statistical analysis, evaluated using Fisher's exact test, showed a p-value of 0.026, indicating a significant difference in the proportion of patients on at least one comedication between the two groups.

Comedications are prescribed less frequently in the group undergoing secondary prophylaxis. A plausible reason for this could be that patients in the secondary group are in a more severe state of illness and, for instance, showed significantly higher serum creatinine levels compared to patients in the primary prophylaxis group (0.82 mg/dL IQR 0.31 vs. 0.90 mg/dL IQR 0.37, p=0.001; evaluated using the Mann-Whitney U test). This difference underlines the potential influence of clinical decision-making processes on treatment approaches, particularly in patients with more advanced liver cirrhosis as often present after an event of variceal bleeding.

**Table-R1: Comparison of Comedication Usage in Primary and Secondary Prophylaxis**

|  | Primary prophylaxis | Secondary prophylaxis | p-value |
| --- | --- | --- | --- |
| Follow-up (months, IQR) | 26.8 (37.9) | 29.2 (56.7) | p=0.782 |
| Bleeding during Follow up (n,%) | 58 (13.2) | 140 (29.2) | p<0.001 |
| Bleeding at one year (n,%) | 17 (3.9) | 38 (7.9) | p=0.012 |
| Death during Follow up (n,%) | 162 (36.8) | 228 (47.5) | p=0.001 |
| Death at one year (n,%) | 60 (13.6) | 92 (19.2) | p=0.026 |
| At least one comedication  (Statin and/or metformin and/or RASi; n,%) | 99 (22.7) | 68 (14.7) | p=0.026 |
| No co-medication (n,%) | 337 (77.3) | 394 (85.3) |  |

Continuous variables are expressed as median with interquartile range (IQR).

Fischer´s exact tests and Mann-Whitney U tests were performed to compare the respective proportions between patients on primary prophylaxis and secondary prophylaxis.

This revised table has been added to the revised manuscript as Table-3 (*Comparison of Co-medication Usage in Primary vs. Secondary Prophylaxis and Child-Pugh A vs. Child-Pugh B/C*). Moreover, these findings have also been included in the revised methods, results and discussion sections of the submitted manuscript:

*“…Comparisons of continuous variables were performed using Student t-test or Mann-Whitney U-test, if applicable. Comparisons of categorical variables were performed using Chi-square or Fisher’s exact test…”*

*“…The median follow-up duration was similar between groups, with primary prophylaxis at 26.8 months (interquartile range: IQR 37.9) and secondary prophylaxis at 29.2 months (IQR 56.7, p=0.782). However, patients in secondary prophylaxis showed a significantly higher incidence of bleeding during follow-up (29.2%) compared to patients in primary prophylaxis (13.2%, p<0.001). Mortality rates also were higher in the secondary group (47.5%) than in the primary group (36.8%, p=0.001). Additionally, one-year outcomes demonstrated significant differences in both bleeding (p=0.012) and mortality rates (p=0.026)…”*

*“…In the analysis of co-medication usage, a significantly higher percentage of patients in the primary prophylaxis group, 22.7%, were found to be on at least one co-medication, in contrast to only 14.7% in the secondary prophylaxis group (p=0.026)… “*

*“…We observed a significantly lower rate of co-medication intake in patients under secondary prophylaxis compared to primary prophylaxis. A plausible reason for this could be that these patients are in a more severe state of illness, and have, for instance, significantly higher serum creatinine levels compared to those in the primary prophylaxis group (0.90mg/dL IQR 0.37 vs. 0.82mg/dL IQR 0.31, p=0.001). This difference underlines the potential influence of clinical decision-making processes on treatment approaches, particularly in the context of patients after a severe event of variceal bleeding…”*

**Table-R2: Comparison of Statin, Metformin, and RAS Inhibitor Usage in Primary and Secondary Prophylaxis**

|  | Primary prophylaxis | Secondary prophylaxis | p-value |
| --- | --- | --- | --- |
| Statin (n,%) | 32 (8.7) | 17 (4.1) | p=0.012 |
| No statin (n,%) | 337 (91.3) | 394 (95.9) |  |
|  |  |  |  |
| Metformin (n,%) | 40 (10.6) | 34 (7.9) | p=0.222 |
| No metformin (n,%) | 337 (89.4) | 394 (92.1) |  |
|  |  |  |  |
| RASi (n,%) | 61 (15.3) | 30 (7.1) | p=0.001 |
| No RASi (n,%) | 337 (84.7) | 394 (92.9) |  |

We have created an additional table, *Table-R2*, for the reviewers' consideration. This table compares the use of statins, metformin, and RASi between patients undergoing primary prophylaxis and undergoing secondary prophylaxis, evaluated using Fisher's exact test. *Table-R2* highlights the differences in comorbidity management between patients in the primary and secondary prophylaxis groups, with significant differences in the use of statins and RAS inhibitors.

**3) Confounders: Some patients in the comed and no-comed groups had ascites, a known major factor related to prognosis and potentially introducing bias. Did the authors perform sub-analyses for patients with and without ascites at baseline, or stratified by CTP-class (e.g., CTP A vs. B/C)? If the number of patients in the respective comed groups is too small, perhaps combining all patients in one group for analysis could be considered.**

We fully agree that ascites is a main discriminator of outcomes in patients with cirrhosis. Thus, we have analyzed the impact of comedications in patients with and without ascites separately, and according to Child-Pugh stages, as indicators of impaired hepatic function. (*see also response to point 4*).

Patients with moderate/severe ascites and those with Child-Pugh stages B/C had received significantly fewer comedications (statin, metformin, or RASi).

**Table-R3: Comparison of Comedication Usage in the presence of ascites**

|  | Ascites | No ascites | p-value |
| --- | --- | --- | --- |
| At least one co-medications  (Statin and/or metformin and/or RASi; n,%) | 79 (15.6) | 84 (22.2) | p=0.014 |
| No comeds (n,%) | 428 (84.4) | 294 (77.8) |  |

**Table-R4: Comparison of Comedication Usage in Child-Pugh A and Child-Pugh B and C**

|  | Child A | Child B/C | p-value |
| --- | --- | --- | --- |
| At least one co-medications  (Statin and/or metformin and/or RASi; n,%) | 70 (27.5) | 62 (12.3) | p<0.001 |
| No comeds (n,%) | 185 (72.5) | 440 (87.6) |  |

We used Table R4 for our manuscript and is included in the revised manuscript as Table-3 (*Comparison of Co-medication Usage in Primary vs. Secondary Prophylaxis and Child-Pugh A vs. Child-Pugh B/C*), which provides Child-Pugh stratification, as we have continued to use it for calculations and risk analyses, as outlined in point 4. Moreover, these findings have also been included in the revised result section of the re-submitted manuscript:

*“…Patients within Child-Pugh stages B/C received significantly fewer co-medications (at least one of the co-medications: Child-Pugh A: 27.5% vs. Child-Pugh B/C: 12.3%, p<0.001)…”*

**4) Comorbidities: One of the major weaknesses of the study, as acknowledged by the authors, is that comeds were not intentionally used for liver purposes, suggesting that patients with comeds were sicker at baseline. This could be a reason why no beneficial effect was observed for patients taking these comeds. At least there should be some statistical adjusting between the comed and not no-comed groups before comparing, or as mentioned before, substratifications of CTP class should be shown and then compare decompensation events such as bleeding or mortality.**

We appreciate this insightful suggestion. Employing a Cox regression model for statistical adjustment for liver disease severity (Child stage) between the comed and non-comed groups to analyze a potential beneficial effect seems a valuable approach to account for this potential confounding factor. Therefore, guided by the reviewer's advice, we compared bleeding and mortality rates but adjusted Child-Pugh score and age in our models. The odds ratio for bleeding risk and mortality was calculated by using a multivariate cox regression model.

We can demonstrate in *Table-R5* that the intake of at least one comedication did not show a significant impact on (re-)bleeding risk, even after adjustment for Child-Pugh score and age.

Interestingly, in *Table-R6,* the mortality rates were not surprisingly higher among patients classified as Child B/C and those of advanced age. However, it was found that the intake of at least one comedication, even after adjustment for age and Child B/C status, did not significantly impact survival outcomes.

Thus, this refined analyses further validated our previous findings and affirmed conclusions of our study.

**Table-R5: Risk of (re-)bleeding using at least one comedication.**

|  | No bleeding | (Re-)bleeding | Multivariate  odds ratio  (95% CI) | p-value |
| --- | --- | --- | --- | --- |
| **Patients (n)** | **710** | **187** |  |  |
| **Age (median, IQR)** | 57.6 (16.2) | 55.5 (17.1) | 0.99 (0.98-1.00) | 0.157 |
| **Child-Stage A vs. B/C (n, %B/C)** | 204/400 (55.4) | 54/90 (45.5) | 1.00 (0.69-1.46) | 0.999 |
| **No comed vs. Comed (n,%Comed)** | 578/132 (18.6) | 153/34 (18.2) | 1.02 (0.63-1.65) | 0.945 |

Percentage (%) refers to the total (n) of patients in the respective subgroup; continuous variables are expressed as median with interquartile range (IQR). The odds ratio for (re-)bleeding risk was calculated by a multivariate binary logistic regression model.

**Table-R6: Risk of death using at least one comedication.**

|  | No death | Death | Multivariate  odds ratio  (95% CI) | p-value |
| --- | --- | --- | --- | --- |
| **Patients (n)** | **529** | **369** |  |  |
| **Age (median, IQR)** | 55.8 (15.9) | 59.7 (16.7) | 1.03 (1.01-1.04) | <0.001 |
| **Child-Stage A vs. B/C (n, %B/C)** | 178/268 (50.7) | 80/249 (67.5) | 2.33 (1.66-3.26) | <0.001 |
| **No comed vs. Comed (n,%Comed)** | 441/88 (16.6) | 290/79 (21.4) | 1.39 (0.92-2.09) | 0.115 |

Percentage (%) refers to the total (n) of patients in the respective subgroup; continuous variables are expressed as median with interquartile range (IQR). The odds ratio for (re-)bleeding risk was calculated by a multivariate binary logistic regression model.

These revised tables have been added as Table-4 to the revised manuscript. Moreover, these findings have also been included in the revised method, result and discussion section of the submitted manuscript:

*“…A Cox regression model was used to assess the effect of variables such as age, Child-Pugh stage, and the use of co-medications on (re-)bleeding and death…”*

*“…Bleeding rates in primary and secondary prophylaxis are shown in Figure-2D and 2E and were not significantly influenced by co-medication status in Year 2. Generally, the intake of at least one co-medication did not show a significant impact on (re-)bleeding risk, even after adjustment for Child-Pugh score and age (odds ratio, OR:* 1.02, *confidence interval 95%CI* 0.63-1.65, *p=*0.945; *see Table-4*)*…”*

*“…Mortality rates in primary and secondary prophylaxis are shown in Figure-3D and 3E and were not significantly influenced by co-medication status in Year 2. Furthermore, for all patients in the multivariate analysis, the intake of at least one co-medication, even after adjustment for age and Child B/C status, did not significantly impact survival outcomes (OR:* 1.39, *95%CI* 0.92-2.09, *p=*0.115, *see Table-4*)*…”*

*“…However, in this multicenter real-life study including many patients, we could not reveal any benefits of additional therapy with metformin, statins, or ACE-I/ARB on the rate of variceal bleeding and mortality.*

*The study could show that the intake of at least one comedication did not show a significant impact either on (re-)bleeding risk or mortality, even after adjustment for a higher Child-Pugh stage. It's also worth mentioning that as shown in Table-4, mortality rates were understandably significantly higher among patients classified as Child B/C and those who were older…”*

**5) Role of NSBB: The role of NSBB in this study is not clear to me. The authors state the numbers of patients receiving NSBB at baseline, but details about continuation/discontinuation after primary or secondary prophylaxis and the duration of use before are missing. It is now widely known that the early use of NSBBs prevents decompensation events. Additionally, it might be worthwhile to explore the synergistic effect of beta-blockers and comeds on the stated endpoints.**

Thank you for commenting on this important point of using NSBB for (re-)bleeding prophylaxis and its impact on mortality. As a very important cofactor for treating portal hypertension, NSBB should be incorporated more extensively into our study. We have created an additional figure to analyze the impact of adding NSBB to individual comedications on bleeding. *Figure-R1* displays Kaplan-Meier curves for bleeding events with NSBB and comeds (statin, metformin, or RASi) versus non-comeds for both primary and secondary prophylaxis. In *Figure-R1*, we demonstrated that in primary and secondary prophylaxis, the synergistic effect of NSBB and all comeds on decompensation such as (re-bleeding) has no significant effect.

Going to detail, in the combination therapy of NSBB and statins, there was no difference in bleeding events observed between primary (HR: 3.37; 95%CI: 0.66-17.39; log-rank p=0.146) and secondary prophylaxis (HR: 0.39; 95%CI: 0.13-1.16; log-rank p=0.216). Additionally, the additional administration of NSBB, either with metformin (primary prophylaxis: HR: 0.81 95%CI: 0.28-2.38; log-rank p=0.703; secondary prophylaxis: HR: 1.35 95%CI: 0.62-2.93; log-rank p=0.203) or RASi (primary prophylaxis: HR: 1.09 95%CI: 0.41-2.88; log-rank p=0.865; secondary prophylaxis: HR: 0.47 95%CI: 0.18-1.18; log-rank p=0.638), did not show any advantage in terms of bleeding events in both primary and secondary prophylaxis.

In our database, we unfortunately only collected precise medication compliance data for the last included patients in our study, aiming for a more accurate assessment of compliance. Among the 37.3% (one-third) of patients for whom we have exact compliance records, 87.2% have consistently taken NSBB over an extended period. Of course, we will mention this point in our limitations section of the discussion.

**Figure-R1: Kaplan-Meier (re-)bleeding curves with NSBB and comedication (statin, metformin or RASi) or non-comedication for primary and secondary prophylaxis**

These revised figures have been added as Figure-5 to the revised manuscript. Moreover, these findings have also been included in the revised result and discussion section of the submitted manuscript:

*“…Bleeding for patients with combined treatment with NSBB and co-medication (statin, metformin or RASi) or non-co-medication in primary and secondary prophylaxis (Figure-5)*

*In Figure-5, patients were subdivided also according to primary and secondary prophylaxis and the impact of combined treatment with NSBB and co-medication on (re-)bleeding was analyzed. In the combination therapy of NSBB and statins, there was no difference in bleeding events observed between primary (HR: 3.37; 95%CI: 0.66-17.39; log-rank p=0.146) and secondary prophylaxis (HR: 0.39; 95%CI: 0.13-1.16; log-rank p=0.216). Additionally, the additional administration of NSBB, either with metformin (primary prophylaxis: HR: 0.81 95%CI: 0.28-2.38; log-rank p=0.703; secondary prophylaxis: HR: 1.35 95%CI: 0.62-2.93; log-rank p=0.203) or RASi (primary prophylaxis: HR: 1.09 95%CI: 0.41-2.88; log-rank p=0.865; secondary prophylaxis: HR: 0.47 95%CI: 0.18-1.18; log-rank p=0.638), did not show any advantage in terms of bleeding events in both primary and secondary prophylaxis…”*

*“… Similarly, the rate of recommended NSBB use was also low in our study, particularly in the context of secondary prophylaxis. This could be due to non-recorded contraindications or intolerance to NSBBs, but also to preferences of the respective treating physicians – as we have previously documented for Austria. However, the impact of the synergistic effect of NSBB to individual co-medications (statin, metformin, or RASi) on bleeding has no significant effect (see Figure-5)…”*

*“…Furthermore, we only have exact compliance records of 37.3% of using NSBB. However, 87.2% have consistently taken NSBB over an extended period, which indicates a high level of compliance…”*

**6) The authors mention that patients in whom co-medication could not be reliably assessed were not considered for this analysis. How many patients fall into this category? It should be stated in the manuscript.**

Thank you for this important comment. We excluded patients from the analysis due to unreliable assessment of comedication. Patients in primary prophylaxis, a total of n=4 patients were not considered due to insufficient information on comeds. In the secondary prophylaxis, n=18 patients were excluded because their comeds could not be sufficiently assessed from their medical history.

For this reason, we added this information to the results section:

*“…The differences in outcomes between primary prophylaxis and secondary prophylaxis, including bleeding during follow-up and death during follow-up, are statistically significant with p<0.001 and p=0.001, respectively.*

*Furthermore, in the primary prophylaxis group, n=99 (22.7%) of patients were on at least one comedication (statin, metformin, or RASi), while in the secondary prophylaxis group was significantly lower with n=68 (14.7%, p=0.026). For this analyzing, patients in primary prophylaxis (n=4) and secondary prophylaxis (n=18) were not considered due to insufficient information on these co-medications…”*

**7) The manuscript lacks p-values in the statistics. If there is no statistical difference between all groups, it should be explicitly stated in the manuscript.**

Thank you for this important note. We also considered this, but since patients can appear in multiple medication categories (leading to double reporting), we did not provide p-values for the baseline characteristics between these 3 groups. To illustrate, we have calculated and included the p-values in Table-R7 and R8. If desired, we are certainly able to incorporate the p-values into our manuscript.

**Table-R7: Characteristics of patients undergoing endoscopic band ligation for primary bleeding prophylaxis according to intake of co-medication of interest**

| Primary prophylaxis (n=440) | No comeds | Statin | Metformin | RASi |  |
| --- | --- | --- | --- | --- | --- |
|  | **337 (76.6%)** | **32 (7.3%)** | **40 (9.1%)** | **61 (13.9%)** |  |
| Age (median, IQR) | 57.2 (15.8) | 66.3 (15.8) | 65.2 (17.6) | 66.3 (13.2) | p<0.001 |
| Sex (m/w, %m) | 225/112 (66.8) | 19/13 (59.4) | 27/13 (67.5) | 33/28 (54.1) | p=0.083 |
| Aetiology of cirrhosis |  |  |  |  |  |
| - Alcohol (n, %) | 169 (50.1) | 14 (43.8) | 17 (42.5) | 27 (44.3) | p=0.124 |
| - Viral hepatitis (n, %) | 71 (21.1) | 5 (15.6) | 8 (20) | 15 (24.6) |  |
| - Mixed (n, %) | 15 (4.4) | 0 (0) | 0 (0) | 0 (0) |  |
| - Other (n, %) | 41 (12.2) | 3 (9.3) | 2 (5) | 2 (3.3) |  |
| - Cryptogenic (n, %) | 41 (12.2) | 10 (31.3) | 13 (32.5) | 17 (27.9) |  |
| Gastric varices (n, %) | 5 (1.5) | 0 (0) | 1 (2.5) | 1 (1.6) | p=0.490 |
| Small varices <5mm (n, %) | 35 (10.4) | 1 (3.1) | 3 (7.5) | 6 (9.8) | p=0.197 |
| Large varices >5mm (n, %) | 290 (86.1) | 30 (93.8) | 36 (90) | 52 (85.2) |  |
| Serum creatinine (mg/dL, IQR) | 0.83 (0.31) | 0.79 (0.40) | 0.74 (0.25) | 0.93 (8.60) | p<0.001 |
| Serum albumine (mg/dL, IQR) | 33.4 (8.1) | 35.9 (12.5) | 36.1 (9.5) | 36 (8.6) | p>0.999 |
| PT (%, IQR) | 59 (23) | 68 (24.5) | 68 (20.5) | 66 (17) | p=0.361 |
| INR (IQR) | 1.3 (0.3) | 1.2 (0.3) | 1.2 (0.2) | 1.2 (0.3) | p=0.186 |
| Serum-bilirubin (mg/dL, IQR) | 1.7 (2) | 1 (1.8) | 1.1 (1.4) | 1.1 (1.1) | p=0.821 |
| MELD (IQR) | 12 (6) | 10 (5) | 9 (5.5) | 10 (5) | p=0.242 |
| Ascites |  |  |  |  |  |
| - mild-moderate (n, %) | 137 (40.7) | 11 (34.4) | 13 (3.3) | 21 (34.4) | p=0.193 |
| - severe/refractory (n, %) | 71 (21.1) | 4 (12.5) | 6 (15) | 6 (9.8) |  |
| Child-Pugh stage |  |  |  |  |  |
| - CPS A (n, %) | 93 (27.6) | 12 (37.8) | 18 (45) | 31 (50.8) | p=0.072 |
| - CPS B (n, %) | 111 (32.9) | 7 (21.9) | 12 (30) | 16 (26.2) |  |
| - CPS C (n, %) | 60 (17.8) | 4 (12.5) | 1 (2.5) | 4 (6.6) |  |
| NSBB use (n, %) | 231 (68.6) | 22 (68.8) | 33 (82.5) | 46 (75.4) |  |
| AST (U/L, IQR) | 46.5 (51) | 36 (33.3) | 36 (14.5) | 41 (22) | p=0.009 |
| ALT (U/L, IQR) | 30 (27) | 26 (17.3) | 28 (19.5) | 25.5 (19.5) | p=0.534 |
| GGT (U/L, IQR) | 90.5 (126.6) | 121 (187.1) | 90 (118) | 108 (93) | p=0.413 |
| Platelets (G/L, IQR) | 89 (75.5) | 106 (105) | 115.5 (108.3) | 109 (74.6) | p=0.530 |
| Median follow-up (months, IQR) | 27.1 (41.4) | 25.4 (26.9) | 24.9 (25.5) | 24.2 (29.9) | p=0.925 |
| Number of endoscopies (per year/IQR) | 2.8 (6.9) | 3 (5.3) | 4 (5) | 3.5 (5.8) | p=0.620 |
| Number of EBL sessions (per year/IQR) | 1.3 (4.3) | 1.7 (2.3) | 12.1 (2.7) | 1.7 (4.8) | p=0.901 |
| First variceal bleeding (n, %) | 45 (13.4) | 2 (6) | 5 (12.5) | 6 (9.8) | p=0.194 |
| OLT (n, %) | 33 (9.8) | 0 (0) | 4 (10) | 2 (3.3) | p=0.190 |
| Death (n, %) | 116 (34.4) | 12 (37.5) | 16 (40) | 27 (44.3) | p=0.280 |

**Table-R8: Characteristics of patients undergoing endoscopic band ligation for secondary bleeding prophylaxis according to co-medications**

| Secondary prophylaxis (n=480) | No comeds | Statin | Metformin | RASi |  |
| --- | --- | --- | --- | --- | --- |
|  | **394 (82.1%)** | **17 (3.5%)** | **34 (7.1%)** | **30 (6.3%)** |  |
| Age (median, IQR) | 53.9 (14.9) | 59.7 (17.6) | 62.3 (16.9) | 61.8 (23.4) | p=0.812 |
| Sex (m/w, %m) | 276/118 (70.1) | 8/9 (47.1) | 26/8 (76.5) | 19/11 (6.3) | p=0.375 |
| Aetiology of cirrhosis |  |  |  |  |  |
| - Alcohol (n, %) | 224 (56.9) | 9 (52.9) | 14 (41.2) | 10 (33.3) | P=0.001 |
| - Viral hepatitis (n, %) | 81 (20.6) | 1 (5.9) | 6 (17.6) | 4 (13.3) |  |
| - Mixed (n, %) | 27 (6.9) | 0 (0) | 3 (8.8) | 0 (0) |  |
| - Other (n, %) | 24 (6.1) | 4 (23.6) | 4 (11.8) | 7 (23.4) |  |
| - Cryptogenic (n, %) | 38 (9.6) | 3 (17.6) | 7 (20.6) | 9 (30) |  |
| Gastric varices (n, %) | 10 (2.5) | 1 (5.9) | 2 (5.9) | 0 (0) | p=0.167 |
| Small varices <5mm (n, %) | 53 (13.5) | 1 (5.9) | 4 (11.8) | 4 (13.3) | p=0.166 |
| Large varices >5mm (n, %) | 302 (76.6) | 15 (88.2) | 29 (85.3) | 24 (80) |  |
| Serum-creatinine (mg/dL, IQR) | 0.90 (0.40) | 0.93 (0.34) | 0.93 (0.31) | 1.10 (0.40) | p=0.018 |
| Serum-albumine (mg/dL, IQR) | 31.5 (9) | 32.8 (12.2) | 32.5 (12) | 37.2 (10.2) |  |
| PT (%, IQR) | 55 (21) | 63 (46.5) | 68 (25) | 63 (31) | p=0.634 |
| INR (IQR) | 1.4 (0.4) | 1.2 (0.7) | 1.2 (0.3) | 1.3 (0.2) | p=0.100 |
| Serum-bilirubin (mg/dL, IQR) | 1.9 (2.1) | 1.4 (1.2) | 1.5 (1.7) | 1.2 (1.1) | p=0.011 |
| MELD (IQR) | 13 (7) | 14 (6) | 11 (5.8) | 12 (5.5) | p=0.014 |
| Ascites |  |  |  |  |  |
| - none (n, %) | 170 (43.1) | 9 (52.9) | 16 (47.1) | 18 (60) | p=0.168 |
| - mild-moderate (n, %) | 144 (36.5) | 8 (47.1) | 11 (32.3) | 10 (33.3) |  |
| - severe/refractory (n, %) | 76 (19.3) | 0 (0) | 5 (14.7) | 2 (6.6) |  |
| Child-Pugh stage |  |  |  |  |  |
| - CPS A (n, %) | 92 (23.4) | 5 (29.4) | 9 (26.5) | 15 (50) | p=0.358 |
| - CPS B (n, %) | 180 (45.7) | 6 (35.3) | 13 (38.2) | 6 (20) |  |
| - CPS C (n, %) | 89 (22.6) | 1 (5.9) | 4 (11.8) | 4 (13.3) |  |
| NSBB use (n, %) | 272 (69) | 14 (82.4) | 25 (73.5) | 18 (60) |  |
| AST (U/L, IQR) | 52 (59) | 57.5 (30.8) | 35.5 (22) | 51 (36.5) | p>0.999 |
| ALT (U/L, IQR) | 32 (28.8) | 36 (19) | 31.5 (30.5) | 31 (21) | p>0.999 |
| GGT (U/L, IQR) | 110 (188.8) | 132 (395.5) | 85 (147.5) | 88 (147.8) | p=0.045 |
| Platelets (G/L, IQR) | 101 (89) | 131 (41) | 91 (62) | 63 (31) | p<0.001 |
| Median FU (months, IQR) | 30.3 (60.9) | 27.3 (23.5) | 25.2 (42.5) | 26.4 (46.8) | p<0.001 |
| Number of endoscopies (per year/IQR) | 1.8 (7.8) | 5.9 (22.4) | 4.4 (10.4) | 2.3 (7) | p<0.001 |
| Number of EBL sessions (per year/IQR) | 1 (6.3) | 4.1 (18.3) | 2.2 (6.8) | 2.2 (4.9) | p<0.001 |
| Variceal Re-bleeding (n, %) | 108 (27.4) | 8 (47.1) | 10 (29.4) | 9 (30) | p=0.313 |
| OLT (n, %) | 41 (10.4) | 1 (5.9) | 1 (2.9) | 2 (6.7) | p=0.274 |
| Death (n, %) | 174 (44.2) | 7 (41.2) | 17 (50) | 19 (63.3) | p=0.461 |

Reviewer 2:

**1) As the authors stated, the major limitation of this study is its retrospective character. Because of that, the groups were not randomized and did not receive statin, metformin, or RASi therapy to treat the complications of liver cirrhosis but rather to treat the patient´s comorbidities.**

In response to the statement highlighting the retrospective character of our study as a major limitation, including the lack of randomization and the fact that statin, metformin, or RAS inhibitor therapy was administered not for liver cirrhosis complications but to treat the patients' comorbidities, I agree with the reviewer's observation. It represents a significant limitation of our work. Given low evidence of data and limited experience regarding the impact of these medications on portal hypertension and its complications in clinical practice, we designed a retrospective analysis of real-life data involving a large cohort of patients. This approach was chosen to provide an initial overview and insights into the subject matter.

**2) In the manuscript, it is not stated if the authors had analysed possible ongoing alcohol abuse, especially in patients with alcoholic liver disease, which increases the risk of variceal bleeding. It would be interesting if the authors could divide patients into cohorts of ongoing alcohol abusers and non-abusers. If the data are not available, a comment on ongoing alcohol use and the impact of cessation of alcohol intake would be valuable.**

Thank you for addressing this important point. We are aware of the lack of data regarding ongoing alcohol abuse. Unfortunately, we only collected follow-up data on alcohol consumption at baseline as the cause of cirrhosis, and not ongoing alcohol abuse. Of course, we will mention this important point in our limitations section of the discussion:

*“…Another important point is that the study primarily focused on collecting data on alcohol consumption at baseline as the identified cause of cirrhosis. Data on ongoing alcohol abuse throughout the follow-up period were not collected…”*
